# A diagnostic protocol designed for determining allergic causes in patients with blood eosinophilia

**DOI:** 10.1186/s40779-017-0124-7

**Published:** 2017-05-23

**Authors:** Jean-François Magnaval, Guy Laurent, Noémie Gaudré, Judith Fillaux, Antoine Berry

**Affiliations:** 1 Department of Medical Parasitology, Faculty of Medicine, Toulouse University, 37 allees Jules-Guesde, Toulouse, France; 2 Purpan Center for Pathophysiology, INSERM UMR U1043, CNRS UMR 5282, Toulouse University, Toulouse, France; 3 Department of Hematology, Toulouse University Hospitals, Toulouse, France; 4 Cancer Institute, Toulouse University and Oncopole, Toulouse, France; 5 Department of Parasitology and Mycology, Toulouse University Hospitals, Toulouse, France; 6 PharmaDev, IRD UMR 152, Toulouse University, Toulouse, France

**Keywords:** Blood eosinophilia, Diagnostic protocol, Classification, Secondary eosinophilias, Allergy, Helminthiases

## Abstract

**Background:**

Blood eosinophilia is a common laboratory abnormality, and its characterization frequently represents a quandary for primary care physicians. Consequently, in France, specialists and particularly hematologists, often must investigate patients who present with blood eosinophilia that often, but not always, occurs because of allergic causes. Both the Departments of Hematology and Parasitology at Toulouse University Hospitals established a collaboration to rule out allergic causes of eosinophilia, particularly helminthiases, prior to initiating more sophisticated investigations.

**Methods:**

Since 2004, the authors employed the same protocol to investigate eosinophilic outpatients who attended the clinic of Parasitology at Toulouse University Hospitals, and they reported the performance of this diagnostic procedure that was designed to be rapid (no hospitalization required) and only moderately expensive.

**Results:**

A total of 406 patients who presented with blood eosinophilia greater than 0.5 (×10^9^, giga cells per litter, G/L) had an allergic etiology in 350 (86.2%) cases. Among the remaining 56 subjects, 17 did not undergo a follow-up and 39 were referred to another specialized department, mostly Hematology. However, only 21 patients attended then were subsequently investigated. Non-allergic causes of eosinophilia, including 3 cases of the lymphoid variant of hypereosinophilic syndrome and 2 cases of myeloproliferative disorder, were identified in 14 patients, whereas 7 remained diagnosed as having idiopathic eosinophilia.

**Conclusion:**

This study underlines the need to investigate patients presenting with even moderate blood eosinophilia. The work-up that was employed appears to be efficient and versatile and may be used by any medical specialist, such as in hematology, infectious disease, or internal medicine departments, who needs to investigate eosinophilic patients and should initially rule out any etiology of allergic eosinophilia.

## Background

Family physicians, as well as internists, pediatricians, and hematologists, can be presented with blood eosinophilia cases. Eosinophilia is a laboratory abnormality that is defined as a permanent increase in the number of circulating eosinophils above a generally accepted threshold of 0.5 G/L [1–4]. A more recent classification of eosinophilia has been proposed by an international working group [5] (Table 1).

**Table 1:** Blood eosinophilia classification, modified from Valent et al. [5]

Classification	Definition
Secondary reactive eosinophilia	Underlying condition/disease in which eosinophils are considered non-clonal cells; eosinophilia considered cytokine driven in most cases
Primary neoplastic eosinophilia	Underlying stem cell, myeloid, or eosinophilic neoplasm, as classified by WHO criteria; eosinophils considered neoplastic cells
Hereditary eosinophilia	Pathogenesis unknown; familial clustering, no signs or symptoms of hereditary immunodeficiency, and no evidence of a reactive or neoplastic condition/disorder underlying eosinophilia
HE of undetermined significance	No underlying cause of eosinophilia, no family history, no evidence of a reactive or neoplastic condition/disorder underlying eosinophilia, and no end-organ damage attributable to eosinophilia

Secondary or reactive eosinophilia may be classified as “allergic” or “non-allergic”, according to the driver of eosinophilopoiesis. Allergic eosinophilias originate from the physiological activation of Th2-driven responses [6] by certain molecules, which are termed “allergens”. Conversely, the etiology of other reactive eosinophilias remains largely unknown, apart from “lymphoid variant of hypereosinophilic syndrome” (L-HES) in which chronic eosinophilia is associated with a minority of circulating T cell clone and high IL-5 levels [7].

Limited data are available regarding the prevalence of blood eosinophilia in the general population of Western countries. In British Columbia, 225 (0.12%) of 195,000 ambulatory outpatients who attended a large laboratory system displayed a blood eosinophil count that exceeded 0.7 G/L [8]. In Southwestern France, among 532 apparently healthy subjects who underwent a health check-up at a local Social Security center, 13 (2.44%) had a count greater than 0.6 G/L [9]. Secondary allergic causes are thought to account for the greatest proportion of eosinophilia cases because other etiologies appear to be uncommon. The overall prevalence of primary eosinophilia is unknown, although a crude estimate of the hypereosinophilic syndrome (HES) incidence in the USA was 0.035 per 100,000 individuals [10].

Consequently, most eosinophilic outpatients who are referred to Hematology or Internal Medicine departments have a secondary allergic cause of eosinophilia. Classically, allergies and atopic status in westernized, industrialized areas, as well as helminthiases in developing countries, are thought to be the most frequent causes of secondary eosinophilia. However, recent increases in travel and migration have occurred over the past four decades, along with improvements in our knowledge of the overall prevalence of helminthiases, such as anisakiasis, strongyloidiasis, or toxocariasis. This has substantially modified the affected patient population. Specialists in Hematology or Internal Medicine, whose first aim is to rule out allergic eosinophilias prior to initiating major investigations, require an enlarged diagnostic evaluation by a cadre of different sub-specialty consultants [4]. Although efficient, this procedure is costly and awkward for patients, who may be tempted to abandon further investigations and then may go on undiagnosed. As of 1990, at Toulouse University Hospitals, physicians from the Hematology and Parasitology Departments met to address this issue. They agreed that determining the most effective diagnostic procedures, along with considerations for patients’ safety, requires a close collaboration between outpatient clinics, which is a main concern for the investigation of eosinophilic subjects. It was decided that ruling out allergic secondary eosinophilia in outpatients should primarily be the responsibility of the Consultation Board of Parasitology. Parasitologists had previously elaborated what they consider to be the best procedure to investigate such patients, at least according to the best medical knowledge and diagnostic possibilities of that time. If no allergic cause of the eosinophilia was found, then the patient was referred to another department, most often Hematology. Because some eosinophilic outpatients were also directly referred by their personal physician to the clinic of the Department of Hematology, the hematologists agreed to first apply all laboratory tests that were included in the procedure described above. Patients who fell into the secondary allergic eosinophilia group were then referred to the Department of Parasitology.

This investigation procedure constantly evolved and was improved but became stable by the mid-2000s. This present article details the finalized version and reports the results it yielded from eosinophilic outpatients who attended the clinic of the Department of Parasitology at Toulouse University Hospitals, where this procedure remains in use.

## Methods

From January 2004 to December 2011, a total of 406 patients (181 females and 225 males) who presented with blood eosinophilia greater than 0.5 G/L were investigated by the same physician (JFM) according to the procedure described below. This cohort included 8 patients who were previously referred to the Department of Hematology by their personal physician, where non-allergic causes of eosinophilia had been ruled out.

### Medical interview

We requested that the patient or the referring physician provided a full medical record to the consultant. This was particularly true for the laboratory results that provided, among other things, eosinophilia kinetics records.

### History of present illness

Symptomatic subjects were interrogated for their date of onset and the types of functional clinical symptoms and complaints.

#### Medical history

In addition to the routine medical and surgical histories, the medical questionnaire probed for the existence of symptoms evocative of an allergic status, such as conjunctivitis, pruritus, sneezing, urticaria, and wheezing. An atopic phenotype could explain, for example, a slight eosinophilia that was permanent or transient during the pollen season. Great attention was paid to the presence of any drug therapy, chronic or recent, because numerous molecules have been recognized as eosinophilia-inducing agents [11]. For example, the frequently used cholesterol-lowering drugs, fibrates and statins, were associated with eosinophilia in 11% of 3,506 long-standing users [12]. Drug addiction was also ruled out, as cocaine or its derivatives may induce both peripheral and pulmonary eosinophilia [13].

#### Social history (occupational and geographical)

The patients’ present and past occupations were considered as highly relevant and were necessarily documented. Professional exposure to certain chemicals may result in chronic eosinophilia. For example, exposure to benzene [14], a chemical that is now intensively used as a component of non-oxygenated unleaded gasoline [15], metal vapors in aluminum plants [16], or cyanoacrylate from industrial glues [17] are well-recognized. Moreover, helminthiases had to be suspected in eosinophilic patients with a history of contact with wet soil or mud, for example, gardeners, laborers, masons, people working in camp sites, or truck farmers. These occupations are at risk for strongyloidiasis, a helminthiasis that is predominantly tropical but is still endemic in pocket areas of Southwestern Europe, particularly France [18], Italy [19], and Spain [20], as well in the Southern United States [21]. People having occupations in contact with soil in developing and/or tropical countries, such as active duty soldiers, or forestry, mining, oil industry, or road building employees, are especially at risk of various helminth infections [22]. Travelers are also affected [23].

Other very important risk factors for helminthiases were not overlooked, principally in patients born and residing in the European Union (EU) or in other westernized countries. Table 2 displays the full content of the questionnaire. Obviously, immigrants were asked about their country of origin. Regarding tourists in at risk areas, we have noticed in our consultative experience that people often forget about countries that they have visited more than a few years ago. We therefore questioned them about the first year they left the EU. This part of the history was of crucial importance because the information gleaned might orientate towards specific laboratory investigations and eventually provide circumstantial evidence in cases where a precise diagnosis might be difficult to establish.

**Table 2:** Questioning assessment of risk factors for helminthiases

*Questionnaire item*
Occupational contact with mud or wet soil (strongyloidiasis)
Rural residence (fascioliasis, toxocariasis, trichinellosis)
Unfenced vegetable garden, and/or presence of stray cats or dogs (toxocariasis)
Hunting (toxocariasis, trichinellosis)
Owning of undewormed pet dogs or cats (toxocariasis)
Food preferences:
. raw/undercooked beef (taeniasis caused by *Taenia saginata*)
. raw/undercooked fish from sea (anisakiasis) or fresh water (infection caused by *Diphyllobothrium sp.*)
. raw/undercooked giblets from calf or lamb (toxocariasis)
. raw/undercooked horse meat (trichinellosis)
. raw/undercooked pork meat (taeniasis caused by *Taenia solium*; trichinellosis)
. raw/undercooked wild boar meat (trichinellosis)
. green salads from vegetable gardens owned by a friend, neighbor, or relative (toxocariasis)
. country green salads, e.g., dandelions or watercress (fascioliasis, toxocariasis)
Travel across/occupation in/developing and/or tropical areas: risk (according to the geographical distribution of helminthiases)
Location of military postings (if overseas deployment, see item above)

#### Physical and medical imaging examinations

Standard physical examinations were performed, which were systematically completed with two basic imaging studies, a chest radiograph (posterior-anterior and lateral views) and an abdominal ultrasonography. This step of the evaluation might yield decisive information. For example, pathognomonic findings, such as *larva currens* in strongyloidiasis, or Calabar swellings in loiasis, represent valuable clues about the allergic origin of the eosinophilia. Conversely, the discovery of enlarged cervical or mediastinal lymph nodes is suspicious for causes of non-allergic eosinophilia.

#### Non-specialized laboratory explorations

##### Blood eosinophil count

The result of a test carried out on the day of clinic attendance, combined with data from a patient’s past medical record, provided definite information about the eosinophilia kinetics. A return to normality, mainly in asymptomatic patients, was a decisive argument to stop an investigation. An undulating curve, sustained over years, was evocative of strongyloidiasis [24] or repeated helminth infections, as could be observed in covert toxocariasis [25] (Table 3).

**Table 3:** Laboratory tests for eosinophilia investigation

I - Non-specialized tests	
	. Total and differential blood count
	. ESR and CRP dosage^a^
	. Test for proteinuria
II - Specialized tests	
	a) Allergy
	. Total IgE titration
	. Global tests detecting IgE specific for food or inhalant allergens
	. Anti-*Aspergillus* IgE assay^b^
	b) Parasitology and Mycology (microscopy examinations)
	. Stool examination including the Baermann’s method
	. Blood examination for microfilariae^c^
	. Skin examination for microfilariae^c^
	. Sputum examination for *Paragonimus sp*. eggs^c^
	. Skin scraping for scabies mites or fungi^d^
	. Urine examination for eggs of *Schistoma haematobium* ^e^
	c) Parasitology and Mycology (immunodiagnostics)
	. Anisakiasis^e^
	. Cystic echinococcosis
	. Fascioliasis^e^
	. Filariases (with Og4C3 detection)^e^
	. Schistosomiasis
	. Strongyloidiasis
	. Toxocariasis
	. Trichinellosis^e^
	. Anti-*A. fumigatus *IgG antibodies

^a^ESR: erythrocyte sedimentation rate; CRP: C-reactive protein

^b^for asthmatic patients and/or presenting with wheezing

^c^for immigrants or travelers only, according to the geographical distribution of relevant helminthiases

^d^if itchy lesions

^e^if relevant risk factors were present

##### Erythrocyte sedimentation rate (ESR) and C-reactive protein (CRP)

Careful attention was paid to the assessment of laboratory inflammatory hallmarks because the values of these tests are usually within the normal range in secondary allergic eosinophilias. However, a pronounced inflammatory syndrome is present during the invasion phase in fascioliasis [26], schistosomiasis japonicum, or mansoni [27], or in lymphatic filariases [28], trichinellosis [29], disseminated strongyloidiasis (hyperinfection syndrome), and visceral *larva migrans* (VLM), which is the major form of toxocariasis [30]. Once the above-cited diseases had been ruled out, the presence of inflammation suggested two possibilities, either the fortuitous association of a common inflammatory disease with a chronic allergic eosinophilia, or an illness inducing primary or secondary non-allergic eosinophilia. In principle, an eosinophilia associated with positive inflammation markers and normal total IgE levels (see “Allergy” section below) was not likely to be allergic in origin [31] (Table 3).

##### Proteinuria detection

Urinalysis was carried out during the visit using a dipstick assay. Usually, proteinuria is absent in allergic eosinophilia, except in urinary schistosomiasis where it is often combined with hematuria, which was detected with multireagent strips. Conversely, proteinuria may accompany rare non-allergic syndromes associated with hypereosinophilia, such as eosinophilic granulomatosis with polyangiitis (EGPA) (Table 3).

#### Specialized laboratory examinations

##### Allergy

***Total immunoglobulin E (IgE) assay***. A rise in total IgE levels in patients chronically infected by worms has been primarily reported in Ethiopian children with ascariasis [32] and in most helminthiases. The pathogenesis of this increase remains partially unknown.

Eosinophilia-inducing diseases other than helminthiases are not associated with an elevated total IgE value, apart from an allergic status in some multi-sensitized patients and, rarely, from L-HES [33] or hyper IgE syndrome [34]. Consequently, a result exceeding 1,000 kIU/L (5-fold higher than the upper limit of the normal range) is mostly an excellent indication of acute or chronic helminth infection (Table 3).

***Global tests for*** **in vitro** ***specific IgE detection***. Atopy is defined by a combination of clinical allergy signs, along with a moderate increase in total IgE counts between 200 and 400 kIU/L [35] and the presence of IgE specific for food or inhalant allergens [36]. A patient meeting these criteria is therefore likely to have mild and/or transient eosinophilia during the pollen season. Therefore, we systematically carried out a global in vitro test detecting specific IgE directed against a mixture of airborne or foodborne allergens [37]. It should be kept in mind that the allergy prevalence is high in westernized countries [38], regardless of age [39], and it is growing in tropical developing countries [40]. Therefore, an atopic status is at risk of being associated with more rare and stringent causes of eosinophilia. Moreover, an atopic status is also present in most L-HES cases [33] (Table 3).

***Detection of specific anti-Aspergillus fumigatus IgE***. This test was performed in patients with a history of asthma and/or patients presenting with wheezing on examination who were at risk of allergic bronchopulmonary aspergillosis [41] (Table 3).

##### Parasitology

Only metazoans (helminths and ectoparasites) and certain fungi, but not protozoans, can elicit eosinophilia. Only microscopy and serology were used in this work-up, as molecular methods for the detection of metazoan parasites were investigational by the time of the study (Table 3).

*Direct microscopic examinations -* For decades, the diagnosis of digestive helminthiases has required multiple stool examinations. To date, microscopy remains the gold standard procedure to ascertain the presence of intestinal parasites; however, it is time-consuming and requires well-trained technicians. Additionally, repeated negative results upset some patients. We therefore examined a single specimen at every patient’s clinic attendance, and we enhanced this step. We combined direct examinations with two different concentration techniques, and a Baermann’s extraction was systematically performed [42]. This method is approximately 4.5 times more efficient than conventional concentration techniques to find *Strongyloides* larvae [43].

The well-known cellulose tape test for the detection of a pinworm infection [42] was very difficult to implement in our clinics or inpatients and was therefore not included in our panel of microscopic investigations.

We looked for microfilariae (mf) in the blood or the skin of immigrants from tropical countries where filariases are encountered, or of patients with a history of working in or traveling across these areas. Night search for lymphatic filariae mf is fraught with difficulties in outpatients. Therefore, by the turn of the 2000s, night blood sampling was replaced with the serological detection of the so-called Og4C3 antigen (see the “Immunodiagnostics” section). Night testing was maintained for patients from areas where *Brugia* sp*.* filariasis is endemic because the Og4C3 assay does not detect this helminthiasis [44]. Snip biopsy is generally considered the gold standard technique for *Onchocerca volvulus* mf detection in the skin. However, in western countries, this test is often considered a surgical procedure; therefore, strict regulations apply. Instead, we employed a scarification method that was reported to be as sensitive as biopsy [45].

A urinalysis was necessary in patients from areas endemic for urogenital schistosomiasis, which now includes Southwestern Europe [46]. We required that the full void, and not just a few milliliters of urine, should be scrutinized, particularly when hematuria is present [42].

Sputum examination for *Paragonimus* sp*.* eggs was carried out in subjects from any wet tropical area who presented with a chronic cough and/or chest radiography abnormalities consistent with tuberculosis [47].

During scabies, classical cutaneous signs are often accompanied by an intense pruritus and an erythematous papular eruption, which may come with peripheral blood eosinophilia [48]. Skin scraping followed by a microscopic examination for mites |42] was therefore performed in any eosinophilic patient complaining of itching.

##### Immunodiagnostics

First, it should be underlined that immunodiagnostic tests are a complement and not a substitute for microscopic methods. When an eosinophilic patient presented at our Consultation Board with a clinical picture that was highly suggestive of a given helminthiasis, for example, facial edemas during a trichinellosis outbreak, only the specific serodiagnosis was requested. Otherwise, we used an assay panel (Table 3) that was primarily designed for patients native to Western Europe, where certain helminthiases are only evaluated if their risk factors are identified. As urogenital schistosomiasis has emerged as an autochthonous disease in Western Europe [46], its specific serodiagnosis has been added in 2014 to this panel. Trichinellosis was checked if relevant risk factors were prevalent, e.g., using wild boar or horse meat or traveling outside the EU, as this zoonosis can present as an asymptomatic chronic eosinophilia, as observed in two outbreaks that occurred in 1998 in the Toulouse area [49]. For immigrants or travelers, we added an immunodiagnostic for filariases, including an ELISA for circulating Og4C3 antigens, which is a test that has dramatically improved the diagnosis of bancroftiasis [50] (Table 3).

##### Mycology

Albeit rarely associated with blood eosinophilia [51], an epidermal mycosis was systematically ruled out by skin scraping of any suspected lesion, particularly foot intertrigo. Moreover, precipitating anti-*A. fumigatus* antibodies, along with specific IgE, were assayed in asthmatic patients at risk of allergic bronchopulmonary aspergillosis (ABPA) [41]. Certain systemic mycoses, such as coccidioidomycosis [52] or disseminated phaeohyphomycosis [53], can be accompanied by blood eosinophilia, but we never had reason to suspect these infections among our attending patients (Table 3).

##### Bacterial and viral infections

A blood eosinophilia has been reported in certain bacterial or viral infections, such as the more advanced forms of HIV [54]. In these situations, eosinophilia lies in the background of a severe clinical and laboratory picture; therefore, these patients usually are referred and then hospitalized in a Department of Infectious Diseases (Table 3).

### Therapeutic challenges

It is a common but false belief that large solitary tapeworms do not induce eosinophilia [55]. However, a moderate increase in the eosinophil count result has been frequently reported in *Taenia saginata*-infected travelers [56, 57]. Eosinophilia has also been observed in *Enterobius*-infected patients who likely harbored a massive worm load [58, 59]. Nevertheless, ruling out an infection caused by *Taenia* sp*.* tapeworms or by pinworms in an eosinophilic subject is often a challenge. Stool examination is hampered by a high rate of false negative results, and specific serodiagnostics are not available.

Taeniasis and enterobiasis are very common autochthonous helminthiases in Europe; therefore, we used therapeutic challenges prior to any further investigation in any asymptomatic eosinophilic patient. We gave an adult patient a single course of praziquantel (15 mg/kg once a day), followed by a 3-day course of albendazole (400 mg once a day). Both drugs are cheap and recognized as safe for these indications at these dosages [60]. The blood eosinophil count was checked 3 weeks after the end of both treatments, and normalization was considered as indirect evidence of taeniasis or enterobiasis. In symptomatic patients, therapeutic challenges were also used when no diagnosis was reached following the completion of our investigations. Praziquantel was administered at the same dosage, but albendazole was then given at 5 mg/kg twice a day for 1 week. Follow up consultations also took place 3 weeks after the therapies ended.

A therapeutic challenge was considered as positive when clinical symptoms-if present-and the eosinophilia disappeared together, or at least substantially decreased.

## Results

Among the 406 patients evaluated, 61.3% (249/406) reported no history of travel outside of the EU or did not have an origin outside the EU. The mean age of these 406 patients was 51.2 years [standard error of the mean (SEM): 1.3 - median: 53 - range: 6–93], and their mean eosinophil count was 1.3 G/L (SEM: 0.1 - median: 0.95 - range: 0.5–19.2).

### Diagnostics provided by the investigation work-up

The allergic causes of blood eosinophilia which were found in 350 patients (86.2%) are listed in Table 4 and detailed, for parasitic diseases, in Table 5. In 45 patients (13 were asthmatic), an atopic status - namely the simultaneous presence of allergy signs and specific IgE against environmental or food allergens - was observed. Due to a negative result of the above-depicted workup, 11 patients were referred to allergology specialists. Then, these 11 patients had their “atopic status” diagnosis confirmed, thus explaining the eosinophilia origin (Table 4).

**Table 4:** Classification of the causes of allergic eosinophilia found in 406 patients

Causes of allergic eosinophilia		Number of positive and negative diagnostics
Parasitology^a, b^		
	a-Strongyloidiasis found by clinical examination (presence of *larva currens)*	2
	b-Various helminthiases detected by microscopy examination	42
	c-Various helminthiases diagnosed by serology^c^	238
Mycology		
	a-Bronchopulmonary aspergillosis	1
	b-Epidermomycosis due to *Trichophyton mentagrophytes* or *T. rubrum*	2
	c-Fungal sinusitis due to *Aspergillus fumigatus*	1
Allergy		
	a-Atopic status only^d^	11
	b-Drug allergy	13
Therapeutic challenges^e^		
	a-Positive result	40
	b-Test providing no information^f^	39
	c-Patient not following-up	17

350 patients were diagnosed as having an allergic cause of eosinophilia in a total of 406 patients

^a^if double positivity (direct examination and serology), only the microscopy result was retained

^b^in cases of multiple helminth infections, only one diagnostic was recorded per patient

^c^if presence of multiple positive results (cross-reactions or multiple infections), then only the most prominent diagnostic was retained per patient

^d^diagnostic retained following advice from specialists in allergology

^e^therapeutic challenges were used prior to any further investigation in any asymptomatic eosinophilic patient. The blood eosinophil count was checked 3 weeks after the end of both treatments, and normalization was considered as indirect evidence of taeniasis or enterobiasis. In symptomatic patients, therapeutic challenges were also used when no diagnosis was reached following the completion of the investigations. Check-up consultation also took place 3 weeks after the therapies ended. A therapeutic challenge was considered as positive when clinical symptoms - if present - and eosinophilia disappeared simultaneously, or at least substantially decreased

^f^no diagnostic reached, further referral to another Department

**Table 5:** Detailed results of laboratory investigations for parasitic infection evaluations in 406 eosinophilic patients

Laboratory investigation		Parasitic infections found as causes of eosinophilia (*n* = 280)
Microscopy (agent)^a,b^		
	Blood	*Loa-loa* (*n* = 2)
		*Mansonella perstans* (*n* = 1)
	Skin	*Onchocerca volvulus* (*n* = 1)
		*Sarcoptes scabiei* (*n* = 2)
	Stools	Hookworms (*n* = 3)
		Hookworms + *Enterobius vermicularis* (*n* = 1)
		Hookworms + *Enterobius vermicularis + Strongyloides stercoralis* (*n* = 1)
		*Enterobius vermicularis* (*n* = 6)
		*Hymenolepis nana* (*n* = 4)
		*Schistosoma mansoni* (*n* = 13)
		*Strongyloides stercoralis* (*n* = 4)
		*Taenia sp.* (*n* = 1)
	Urines	*Schistosoma haematobium* (*n* = 3)
Immunodiagnosis (infection)^a,c^		
		*Anisakiasis* (*n* = 19)
		*Cystic echinococcosis* (*n* = 2)
		*Filariases (except bancroftiasis)* (*n* = 12)
		*Bancroftiasis (positive Og4C3 detection)* (*n* = 4)
		*Schistosomiasis* (*n* = 8)
		*Strongyloidiasis* (*n* = 29)
		*Toxocariasis* (*n* = 160)
		*Trichinellosis* (*n* = 4)

280 out of 406 patients displayed a positive result of laboratory investigations for parasitic infection

^a^If double positivity (direct examination and serology), then only the microscopy result was retained

^b^if multiple helminthes infections were noted, then only one diagnostic was recorded per patient

^c^if multiple positive results were noted (cross-reactions or multiple infections), then only the most prominent diagnostic was retained per patient

**Fig. 1:**
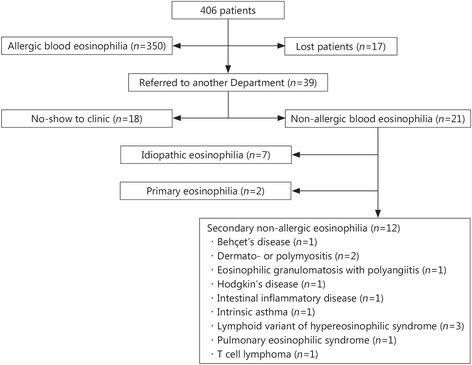
Repartition of the diagnoses made in 406 eosinophilic patients

Figure 1 shows the general repartition of the patients according the diagnostics that were or were not made.

### Cost-effectiveness of the investigations

An assessment of the cost-effectiveness of this work-up indicated that the overall cost of the investigations, as reimbursed by the French National Health Insurance, was moderate. The cost ranged from 412 € for an asymptomatic patient without any risk factor or history of traveling outside of the EU to a maximum of 582 €. Additionally, the cost-effectiveness of the procedure was improved by using therapeutic challenges in asymptomatic patients. If positive, this test ruled out the presence of taeniasis or pin-worm infections for about 19 € (including the total and differential blood count at the check consultation). Moreover, this diagnostic approach, as implemented at a specialized outpatient clinic, was fast because no hospitalization was required, and all laboratory results were generally available within a (maximum) 2-week period.

### Diagnostic recommendations drawn from the study

The above-depicted diagnostic approach can be applied globally, allowing for modifications according to local idiosyncrasies, particularly those concerning the epidemiology of helminth infections.

#### Diagnostic algorithm for the investigation of a blood eosinophilia

**Fig. 2:**
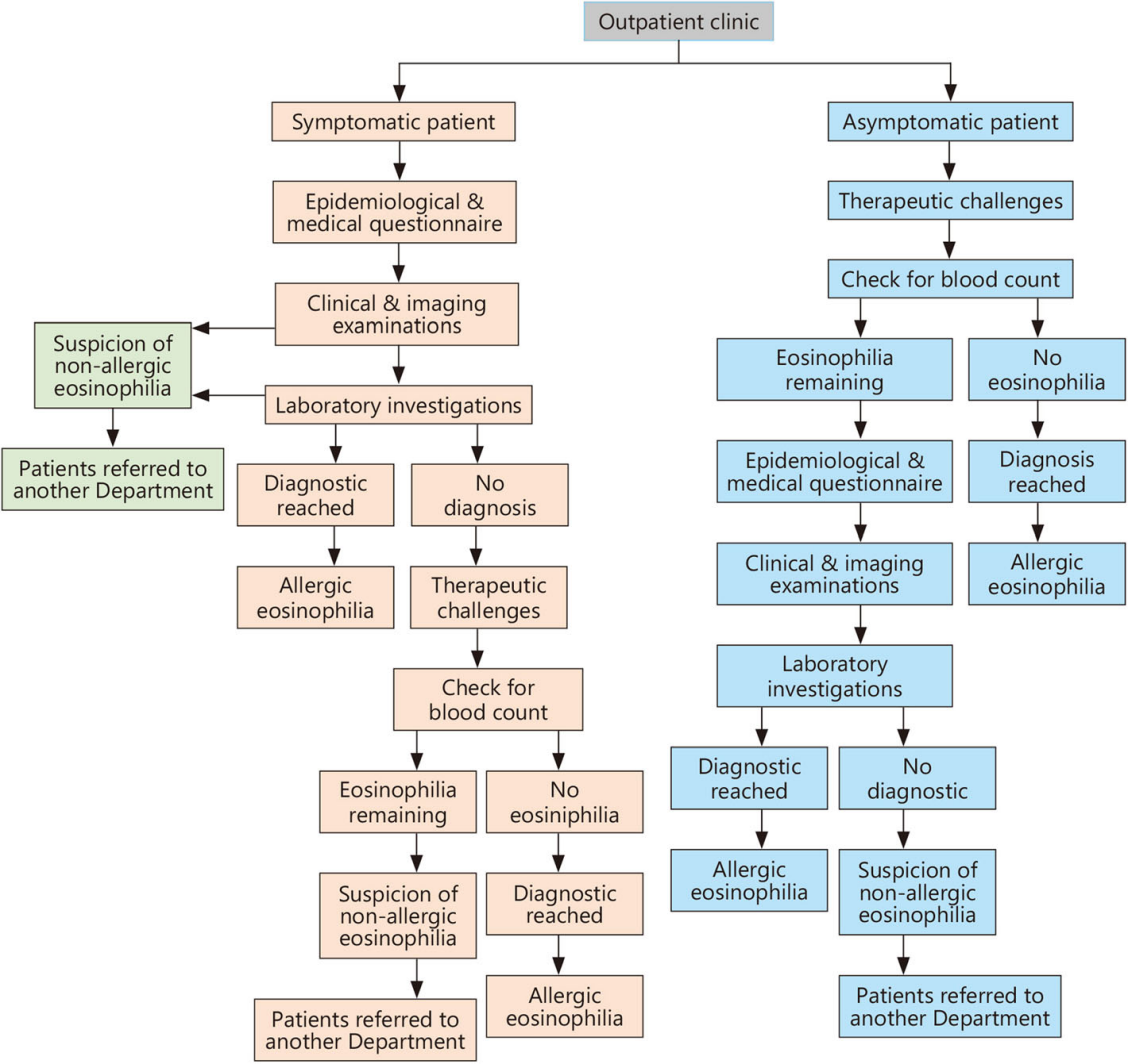
Proposed algorithm for seeking allergic causes in eosinophilic patients

Figure 2 provides a graphical description of the main steps and outputs in the core of the above-depicted work-up.

#### Special consideration for certain helminthiases

The results of parasitology examinations displayed in Table 5 indicate a high rate for three helminthiases, which is a phenomenon that should be explained.

Studies carried out over the last 15 years regarding anisakiasis have unveiled a new spectrum of this zoonosis. It may be accompanied by clinical and laboratory signs of allergy, including moderate eosinophilia [61, 62]. Anisakiasis has acquired the status of an “emergent disease” [63]. Since 2005, we have checked for specific anti-*Anisakis* IgE and IgG4 levels (ImmunoCap®, Phadia, Uppsala, Sweden) in 187 eosinophilic patients who reported a food habit of fresh sea fish or sea food (cephalopods). Anisakiasis serology was the only test that was found to be positive in 19 (10.2%) patients. Their eosinophilia waned following a 7-day course of albendazole combined with appropriate dietary instructions, including no intake of fresh sea fish or cephalopods (cuttlefishes or squids).

Strongyloidiasis is unique among the helminthiases as it is long standing and possibly permanent because of the presence of an autoinfection pattern in the lifecycle of this parasite [64]. Humans become infected when they contact free-living infective larvae that are present in soil polluted with feces. Moreover, strongyloidiasis also has a feature that makes it potentially lethal. This occurs when the autoinfection cycle becomes sharply exacerbated because of a concomitant debilitating disease or the administration of immunosuppressive drugs, particularly corticosteroids. This is termed “hyperinfection syndrome” or “disseminated strongyloidiasis”. As even a brief vacation trip in a developing/tropical area a long time ago may be a source of infection, strongyloidiasis must be suspected in any eosinophilic patient. Fecal examination, even using the Baermann’s extraction or Arakaki’s gelose plate methods, can still miss a considerable percentage of true infections. However, the use of immunodiagnosis has considerably increased the detection rate because commercial test kits for ELISA have proven to be rather sensitive (range: 83.8–96.4%) and specific (range: 95.9–100.0%) [65].

Within the previous 25 years, toxocariasis has evolved in status from a serious medical rarity to that of a trivial, often benign, but eosinophilia-inducing disease. The wide availability of a sensitive and specific immunodiagnostic method, which is known as the TES-ELISA and is based on the use of excretory-secretory antigens from *Toxocara canis* larvae (TES-Ag), has clarified the situation of this zoonosis. Since 1979, numerous seroepidemiological surveys using TES- ELISA have been carried out worldwide and provided concordant results. In urban areas of western countries, the seroprevalence of toxocariasis ranges from 2.5 to 5% in adults and reached 37–44% in rural areas. This rate was found to be far higher in developing countries and peaked at 86% (children) and 92% (adults) in wet tropical islands (reviewed by Fillaux et al. [66]). Most of these positive results are thought to correspond to self-cured mild infections that have left behind residual antibodies that persisted for years [67]. Consequently, it is sometimes a diagnostic quandary to prove that toxocariasis is the etiology of an eosinophilia.

Due to the emergence of urogenital schistosomiasis in Western Europe (Southeastern Corsica [46]), specific microscopy and serological examinations should now be systematically carried out in any European patient who exhibits hematuria and/or blood eosinophilia, even with a lack of any history of travel outside Europe.

## Discussion

This work-up was designed to rule out secondary allergic etiologies in eosinophilic outpatients. Its performance level appears to be high, as it provided an 86.2% positive diagnosis rate. By contrast, investigations carried out in 225 North American outpatients who exhibited blood eosinophilia greater than 0.7 G/L reached a diagnosis in 74% of cases [8]. However, parasitic infections were only tested for in 132 patients, and allergy was retained as the major cause of eosinophilia. This finding was questionable because allergy is a frequent status that may therefore be associated with another cause of eosinophilia. For example, in an English series of eosinophilic travelers, 76 patients had a recorded history of atopy, among whom 41 also had a helminth that was identified [68].

Generally, more complete testing was performed in travel clinics when eosinophilic patients were characterized. Schulte et al. [56] investigated 689 German eosinophilic travelers and reached a diagnosis in 36% of these subjects, although these authors included 56 cases (8.1%) of protozoan infections that are not considered to be eosinophilia inducers. Albeit the reported work-up was very close to ours, the finding rate was significantly lower (*χ* ^2^: 408.9; *P* <0.00000). Among 82 Israeli eosinophilic travelers, 58 (70.7%) were found to have an allergic cause of eosinophilia [69]. Additionally, the diagnostic rate differed significantly from ours (*χ*2: 11.89; *P* <0.00056). Globally, their work-up was similar to that depicted in our report; however, the authors underlined that the diagnostic process was distributed between different departments and time periods. Among 261 eosinophilic subjects presenting at an English tropical disease outpatient clinic, 167 (64%) patients were found to harbor a helminth infection and 57 (22%) patients remained undiagnosed [68]. It was not possible to compare these results with those from the present study, as the described work-up only aimed to rule out mostly tropical helminthiases.

### Study limitations

A recruitment bias could be suspected, as a high rate of parasitic infections was found in patients referred to the Department of Parasitology. An explanation could be that general practitioners and specialists in the study area were intensively taught during post-graduate sessions about eosinophilias. Therefore, they were particularly motivated to have their eosinophilic patients investigated. Consequently, 398 out of 406 patients were outpatients, among whom 292 (73.4%) had been referred to our Consultation Board because their personal physician had detected eosinophilia on a routine blood count and 106 had been sent by various specialists. The remaining 8 patients were from the clinic of the Department of Hematology.

It was therefore surprising to observe that a substantial proportion, i.e., 14 (3.3%) of 398 patients, were subsequently diagnosed as having a major, sometimes life-threatening cause of eosinophilia related to primary or non-allergic secondary etiologies, including 7 (1.76%) cases of hematologic malignancies. However, a recent study of 2,642 patients who presented with an eosinophil count greater than 0.5 G/L reported that 25 (1.14%) patients were diagnosed as having a hematologic malignancy, a finding that did not significantly differ (*χ* ^2^: 3.45; *P*: 0.06) from our present findings [70]. One possible explanation for these results is that patients who present with a disease that elicits a non-allergic eosinophilia develop rather rapidly pronounced clinical signs, whereas most allergic causes of eosinophilia, particularly helminthiases, often induce mild or unrecognized clinical symptoms; therefore, these subjects go undiagnosed. For example, strongyloidiasis can remain practically silent for decades [71]. One must remember, however, that visceral involvement in HES may be absent for years, only to be suddenly revealed in a dramatic manner [72].

### Caveat

It should be underlined that the high prevalence of allergic causes in eosinophilic patients makes the simultaneous presence of a primary or secondary non-allergic etiology possible. This represents a harmful diagnostic pitfall and emphasizes the need for a close collaboration among specialists.

## Conclusion

The most striking finding from this French series of eosinophilic patients was the high toxocariasis rate (39.4%). This zoonosis is now considered to be a major cause of chronic eosinophilia in the study area and probably in other westernized countries. Strongyloidiasis was the second leading etiology (10.4%), with 13 cases diagnosed by fecal examination and 29 by serology. This finding represents a major argument to systematically include both Baermann’s extraction and a specific immunodiagnosis in a panel of routine laboratory methods when investigating a blood eosinophilia case. Our study also highlights the risk of hyperinfection syndrome in eosinophilic patients who undergo immunosuppressive therapy. Surprisingly, anisakiasis was the third most common diagnosis. This finding was the result of the efficiency of epidemiologic questioning, which was designed to identify a broad range of risk factors for eosinophilia-inducing illnesses in these French patients: this approach is rapid (requiring less than 10 min per patient), cheap, and clearly effective.

Accordingly, the detection of an allergic etiology, including helminthiases, is foremost in blood eosinophilia investigations. Given that an eosinophilic outpatient may attend any of the Department of Medicine Clinics for their first visit, the versatility of this present protocol, which can be implemented by most consulting physicians in hematology, clinical immunology, infectious diseases, or internal medicine, appears to be attractive.

## Abbreviations

ABPA: Allergic bronchopulmonary aspergillosis
CRP: C-Reactive Protein
EGPA: Eosinophilic granulomatosis with polyangiitis
ESR: Erythrocyte sedimentation rate
EU: European Union
G/L: Giga cells per liter
HES: Hypereosinophilic syndrome
IgE: Immunoglobulin E
IL 5: Interleukin 5
kIU/L: Kilo International Units per liter
L-HES: Lymphoid variant of hypereosinophilic syndrome
mf: Microfilariae
Th2: T helper cell type 2
